# Supplementary material for: Modified World Health Organization (WHO) Tunnel Test for Higher Throughput Evaluation of Insecticide-Treated Nets (ITNs) Considering the Effect of Alternative Hosts, Exposure Time, and Mosquito Density
Source: Insects. 2022 Jun 21;13(7):562. doi: 10.3390/insects13070562 (PMC9323354; doi:10.3390/insects13070562)
Supplement: Supplementary file 1 [file insects-13-00562-s001.zip › insects-1702355-supplementary.pdf]

Supplementary material

Tables

Table S1. Mean percentage mortality and 95% confidence interval (95% CI) for the negative control, Interceptor®G2, and Interceptor® at 24 hours post exposure (M24), mortality at 72 hours post exposure (M72), and blood feeding success (BFS) or blood feeding inhibition (BFI) of resistant *Anopheles arabiensis* with 12 hours of exposure time for rabbit, membrane and human arm; and 1 hour exposure time for membrane and human arm in the WHO tunnel test. The negative control thresholds for the WHO tunnel test are blood feeding success ≥ 50% and M24 ≤ 10%.

**Assay**	**Blood feeding success**	**Mortality**	
	% BFS (95%CI)	% M24 (95%CI)	% M72 (95%CI)
**Control**			
12hr-Rabbit	64.8 (51.2 - 78.3)	3.8 (0.8 - 6.8)	7.7 (5.1 - 10.3)
12hr-Membrane	22.8 (10.4 - 35.1)	6.8 (5.9 - 7.6)	8.9 (8.3 - 9.5)
12hr-Human arm	74.4 (67.9 - 80.8)	6.4 (4.9 - 7.8)	11.7 (9.0 - 14.4)
1hr-Membrane	18.1 (12.5 - 23.6)	6.8 (5.0 - 8.7)	12.3 (9.1 - 15.4)
1hr-Human arm	65.5 (50.9 - 80.2)	7.2 (6.4 - 8.0)	9.4 (8.3 - 10.5)
	**Blood feeding inhibition**	**Mortality**	
**Treatment**	% BFI (95%CI)	% M24 (95%CI)	% M72 (95%CI)
**Interceptor®**			
12hr-Rabbit	93.4 (89.0 - 97.8)	58.1 (36.6 - 79.5)	69.9 (50.5 - 89.2)
12hr-Membrane	97.0 (94.3 - 99.7)	49.7 (34.9 - 64.6)	65.6 (52.0 - 79.2)
12hr-Human arm	52.8 (44.3 - 61.2)	35.2 (23.0 - 47.3)	43.0 (29.9 - 56.0)
1hr-Membrane	96.0 (93.3 - 98.7)	24.7 (17.0 - 32.4)	38.9 (26.5 - 51.2)
1hr-Human arm	58.2 (48.7 - 67.7)	20.3 (17.7 - 22.8)	31.1 (26.1 - 36.1)
**Interceptor®G2**			
12hr-Rabbit	86.5 (81.8 - 91.1)	41.3 (25.5 - 57.1)	62.3 (51.2 - 73.5)
12hr-Membrane	95.4 (92.3 - 98.5)	43.3 (26.3 - 60.3)	68.8 (52.4 - 85.1)
12hr-Human arm	44.1 (37.3 - 50.9)	23.4 (16.5 - 30.4)	31.7 (24.0 - 39.4)
1hr-Membrane	98.8 (97.7 - 99.9)	44.1 (21.4 - 66.8)	55.8 (34.7 - 76.8)
1hr-Human arm	53.8 (45.9 - 61.7)	11.4 (9.1 - 13.6)	22.3 (18.3 - 26.2)

Table S2. Mean percentage mortality and 95% confidence interval (95% CI) for the negative control, susceptible *Anopheles gambiae* with Interceptor®, resistant *Anopheles arabiensis* with Interceptor®G2 at 24 hours post exposure (M24), mortality at 72 hours post exposure (M72) and blood feeding success (BFS) or blood feeding inhibition (BFI) with rabbit bait and a density of 50 or 100 mosquitoes in the WHO tunnel test. The negative control thresholds for the WHO tunnel test are blood feeding success ≥ 50% and M24 ≤ 10%.

**Assay**	**Blood feeding success**	**Mortality**	
	% BFS (95%CI)	% M24 (95%CI)	% M72(95%CI)
**Control**			
100-Mosquito	83.0 (73.6 - 92.4)	4.7 (3.1 - 6.3)	7.8 (6.2 - 9.4)
50-Mosquito	68.3 (56.3 - 80.3)	8.1 (3.5 - 12.8)	10.2 (7.2 - 13.1)
	**Blood feeding inhibition**	**Mortality**	
**Treatment**	**% BFI** (95%CI)	% M24 (95%CI)	% M72(95%CI)
**Susceptible *An. gambiae with* Interceptor®**			
**Overall**			
100- Mosquito	94.2 (91.8 - 96.6)	98.3 (97.5 - 99.1)	99.1 (98.6 - 99.6)
50- Mosquito	90.9 (88.4 - 93.4)	98.4 (97.5 - 99.3)	99.6 (99.3 - 99.9)
**Unwashed**			
100- Mosquito	93.9 (89.7 - 98.0)	98.2 (96.9 - 99.5)	99.1 (98.2 - 99.9)
50- Mosquito	90.4 (87.1 - 93.6)	98.9 (98.1 - 99.8)	99.7 (99.4 - 100.1)
**Washed 20x**			
100- Mosquito	94.5 (91.9 - 97.2)	98.4 (97.6 - 99.3)	99.2 (98.6 - 99.7)
50- Mosquito	91.4 (87.5 - 95.4)	97.9 (96.2 - 99.5)	99.5 (99.0 - 99.9)
**Resistant *An. arabiensis with* Interceptor®G2**			
**Overall**			
100- Mosquito	87.5 (84.0 - 91.1)	51.8 (41.9 - 61.7)	73.9 (66.7 - 81.2)
50- Mosquito	81.7 (76.8 - 86.7)	45.1 (40.7 - 49.6)	70.0 (67.0 - 73.1)
**Unwashed**			
100- Mosquito	87.5 (82.2 - 92.7)	54.1 (40.3 - 67.9)	74.1 (65.0 - 83.2)
50- Mosquito	82.4 (76.5 - 88.4)	45.8 (41.4 - 50.2)	71.2 (67.8 - 74.7)
**Washed 20x**			
100- Mosquito	87.6 (82.7 - 92.5)	49.6 (35.0 - 64.1)	73.8 (62.2 - 85.3)
50- Mosquito	81.1 (73.0 - 89.1)	44.4 (36.5 - 52.3)	68.8 (63.8 - 73.9)

Table S3. Mean percentage mortality and 95% confidence interval (95% CI) for the negative control, resistant *Anopheles arabiensis* with Interceptor ® or Interceptor®G2 at 24 hours post exposure (M24), mortality at 72 hours post exposure (M72) and blood feeding success (BFS) or blood feeding inhibition (BFI) with rabbit bait and a density of 100 mosquitoes (rabbit-100) or membrane bait and a density of 50 mosquitoes (membrane-50) in the WHO tunnel test. The negative control thresholds for the WHO tunnel test are blood feeding success ≥ 50% and M24 ≤ 10%.

**Assay**	**Blood feeding success**	**Mortality**	
	% BFS (95%CI)	% M24 (95%CI)	% M72(95%CI)
**Control**			
100-Rabbit	64.5 (54.8 - 74.2)	3.9 (1.8-6.1)	7.9 (6.0-9.7)
50-Membrane	25.9.1 (21.8 - 30.1.2)	8.7 (6.6-10.7)	14.6 (12.8-16.5)
	**Blood feeding Inhibition**		**Mortality**
**Treatment**	% BFI (95%CI)	% M24 (95%CI)	% M72(95%CI)
**Interceptor®**			
**Overall**			
100-Rabbit	98.3 (97.5 - 99.1)	56.4 (45.3 - 67.6)	69.4 (60.0 - 76.6)
50-Membrane	98.8 (98.3 - 99.2)	52.5 (45.6 - 59.4)	73.0 (66.9 - 79.1)
**Unwashed**			
100-Rabbit	97.6 (96.1 - 99.0)	52.0 (41.8 - 62.2)	65.5 (57.6 - 71.1)
50-Membrane	98.6 (98.0 - 99.3)	45.8 (36.3 - 55.2)	68.8 (58.5 - 79.1)
**Washed 20x**			
100-Rabbit	87.5 (84.0 - 91.1)	51.8 (41.9 - 61.7)	68.9 (62.7 - 70.2)
50-Membrane	98.9 (98.3 - 99.6)	59.3 (50.2 - 64.4)	73.2 (66.1 - 76.3)
**Interceptor®G2**			
**Overall**			
100-Rabbit	81.7 (76.9 - 86.6)	45.1 (40.7 - 49.6)	73.9 (66.7 - 81.1)
50-Membrane	97.7 (96.8 - 98.7)	56.6 (50.0 - 63.1)	82.5 (78.5 - 86.6)
**Unwashed**			
100-Rabbit	87.5 (82.2 - 92.7)	54.1 (40.3 - 67.9)	74.1 (65.0 - 83.2)
50-Membrane	96.8 (95.2 - 98.4)	52.1 (42.7 - 61.6)	80.1 (73.0 - 87.1)
**Washed 20x**			
100-Rabbit	87.6 (82.7 - 92.5)	49.6 (35.0 - 64.1)	73.8 (62.2 - 85.3)
50-Membrane	98.7 (97.8 - 99.5)	61.0 (52.2 - 69.8)	85.0 (81.0 - 89.0)

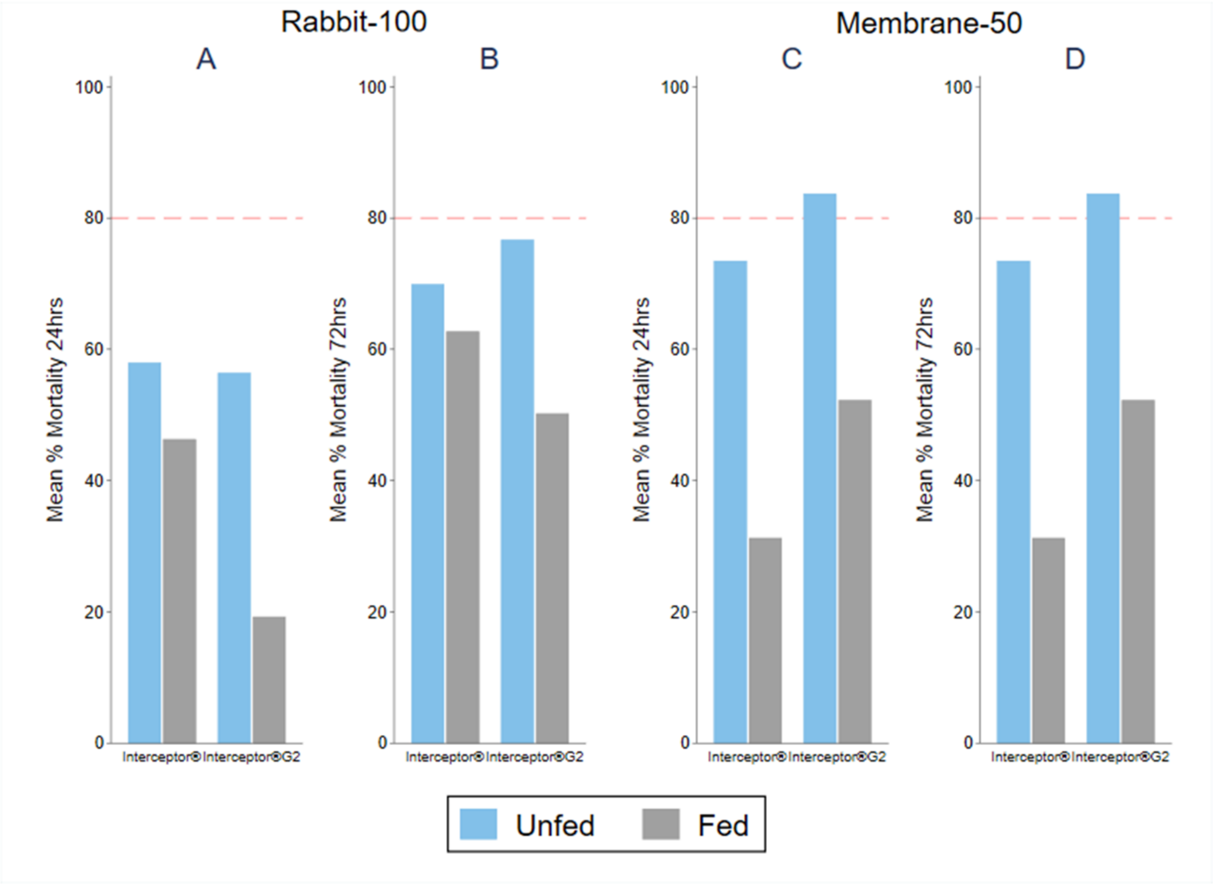

Figure S1: Mean percentage mortality at (A) 24-hours and (B) 72-hours with rabbit-100, (C) at 24-hours and (D) 72-hours with membrane-50, of blood fed and unfed resistant *Anopheles arabiensis* in the WHO tunnel test. Red dashed line depicts WHO mortality threshold of ≥ 80% mortality.

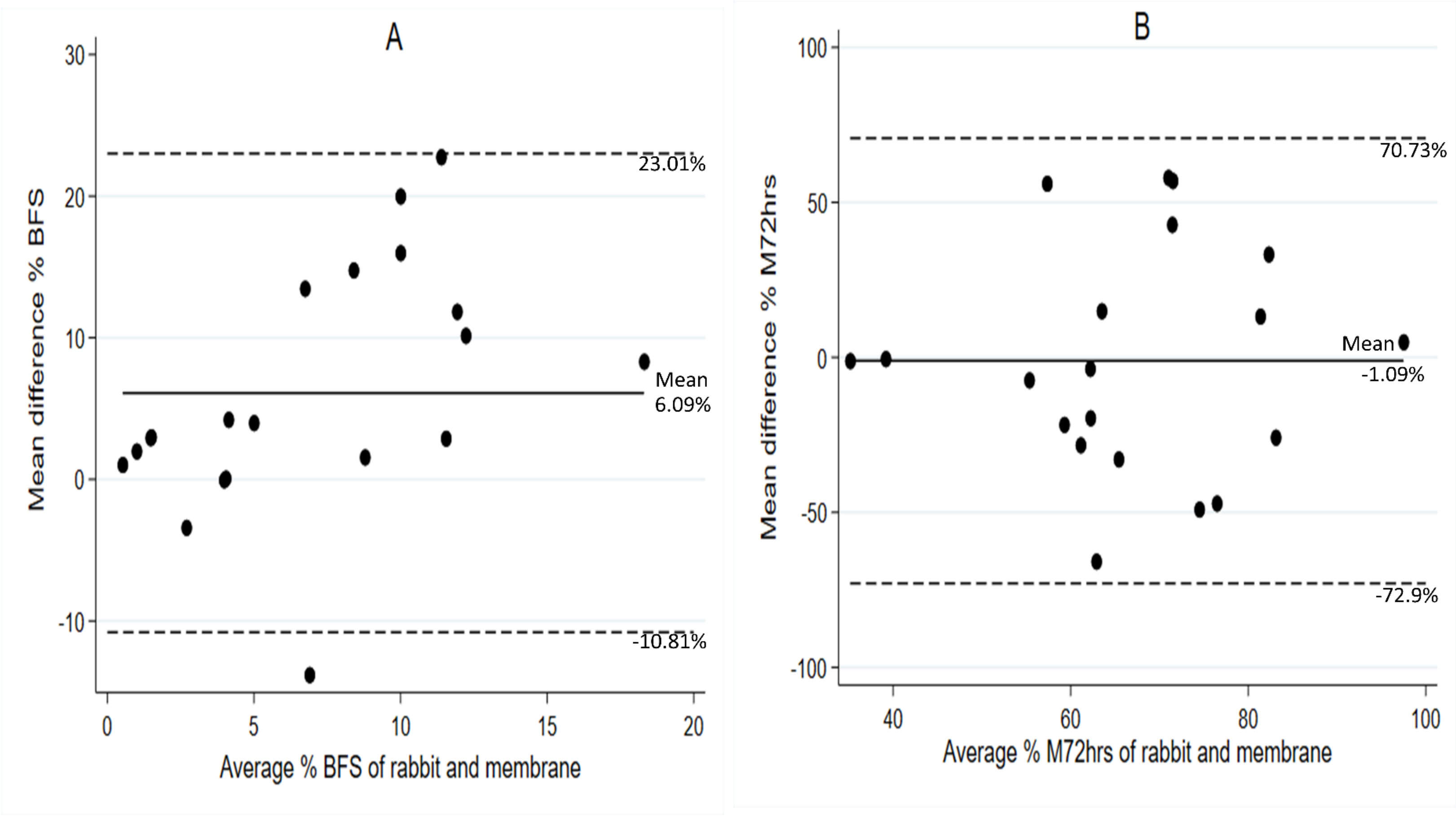

Figure S2: Bland and Altman plot of (A) blood feeding success (BFS) and (B) 72-hours mortality (M72) for Interceptor® and Interceptor®G2 with rabbit or membrane bait with a density of 100-pyrethroid resistant *Anopheles arabiensis* and 12-hour exposure time in the WHO tunnel test. The average value for both methods is plotted on the x-axis and the mean difference between methods on the y-axis. The solid line in the middle shows mean difference with 95% confidence interval of the mean difference represented by the dashed lines.

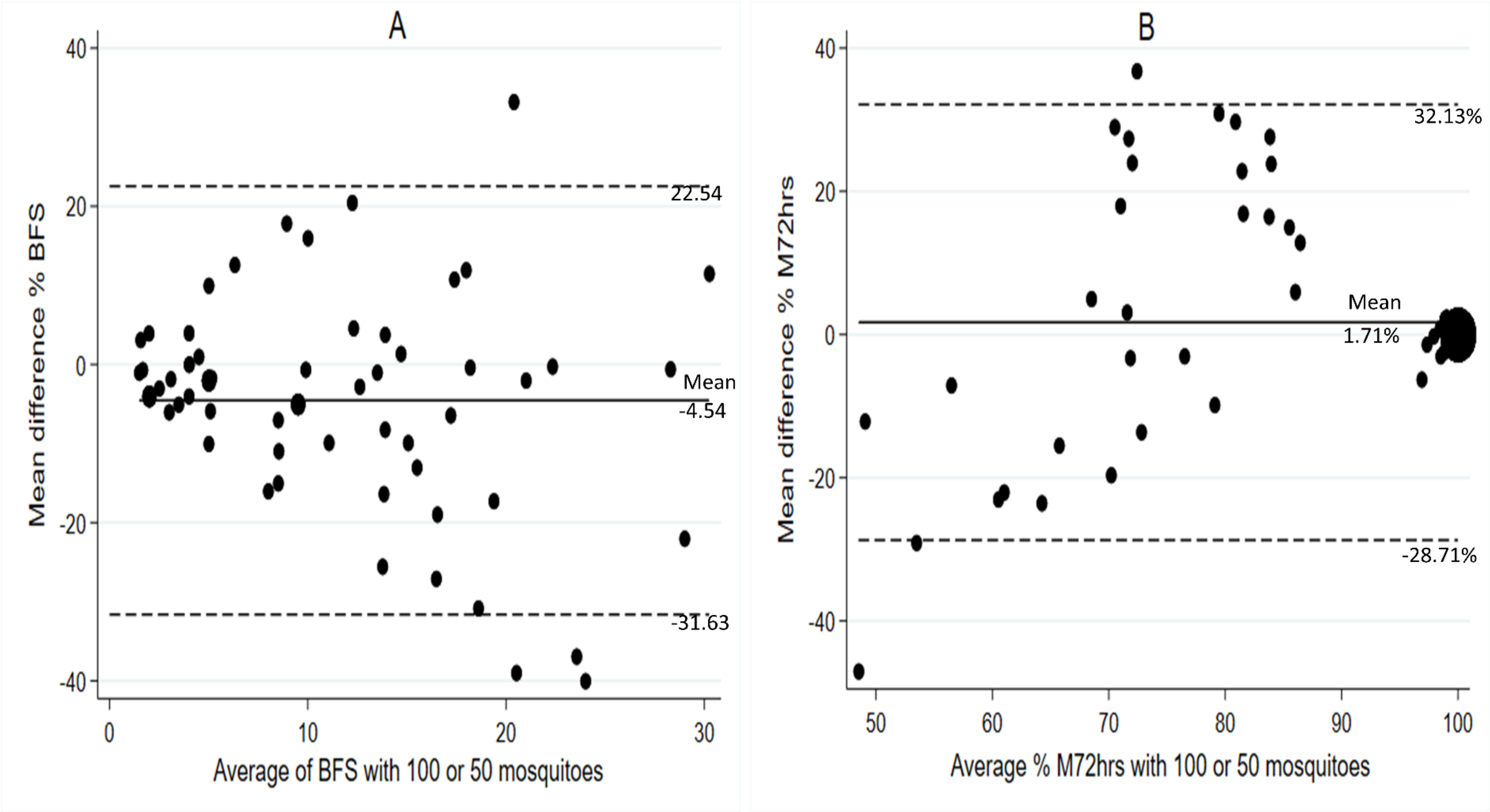

Figure S3: Bland and Altman plot of (A) blood feeding success (BFS) and (B) 72-hours mortality (M72) for Interceptor® with susceptible *Anopheles gambiae* and Interceptor®G2 with resistant *Anopheles arabiensis* using rabbit bait and a density of either 100 or 50 mosquitoes and a 12-hour exposure in the WHO tunnel test. The average value for both densities is plotted on the x-axis and the mean difference between densities on the y-axis. The solid line in the middle shows mean difference with 95% confidence interval of the mean difference represented by the dashed lines.

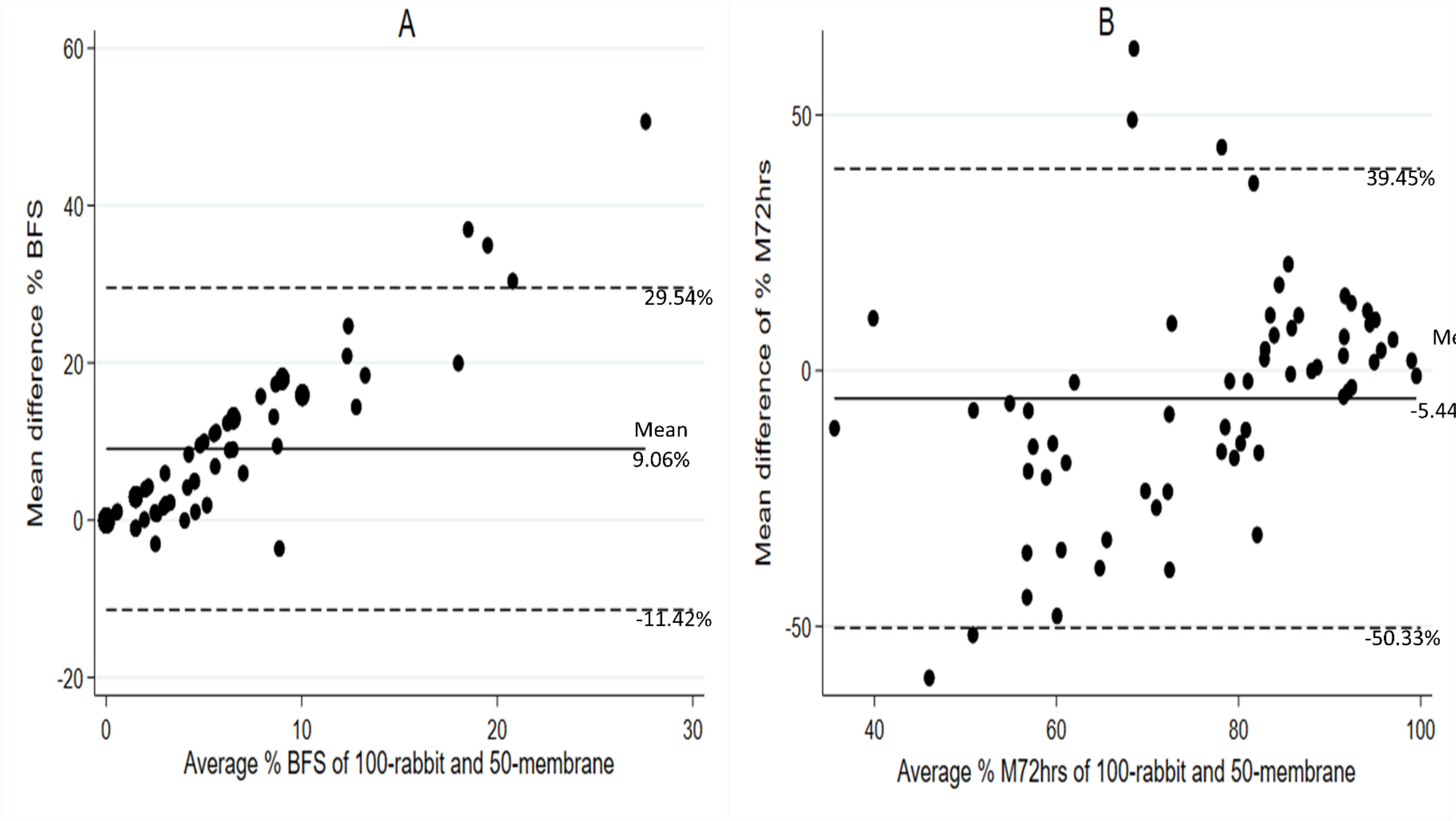

Figure S4: Figure S4: Bland and Altman plot of (A) blood feeding success (BFS) and (B) 72-hours mortality (M72) for Interceptor® and Interceptor®G2 with resistant *Anopheles arabiensis* using rabbit bait and a density of 100 mosquitoes or membrane bait and a density of 50 mosquitoes with a 12-hour exposure time in the WHO tunnel test. The average value for both densities is plotted on the x-axis and the mean difference between densities on the y-axis. The solid line in the middle shows mean difference with 95% confidence interval of the mean difference represented by the dashed lines.
